# Precise enhancement quantification in post-operative MRI as an indicator of residual tumor impact is associated with survival in patients with glioblastoma

**DOI:** 10.1038/s41598-020-79829-3

**Published:** 2021-01-12

**Authors:** Alonso Garcia-Ruiz, Pablo Naval-Baudin, Marta Ligero, Albert Pons-Escoda, Jordi Bruna, Gerard Plans, Nahum Calvo, Monica Cos, Carles Majós, Raquel Perez-Lopez

**Affiliations:** 1 Radiomics Group, Vall d’Hebron Institute of Oncology (VHIO), 117 Natzaret, 08035 Barcelona, Spain; 2 Department of Radiology, Institut de Diagnòstic Per La Imatge (IDI), Bellvitge University Hospital, Barcelona, Spain; 3 Neuro-Oncology Unit, Institut d’Investigació Biomèdica de Bellvitge (IDIBELL), Barcelona, Spain; 4 Department of Neurology, Bellvitge University Hospital, Barcelona, Spain; 5 Department of Neurosurgery, Bellvitge University Hospital, Barcelona, Spain; 6 Department of Radiology, Vall d’Hebron University Hospital, Barcelona, Spain

**Keywords:** Neurology, Oncology, Mathematics and computing

## Abstract

Glioblastoma is the most common primary brain tumor. Standard therapy consists of maximum safe resection combined with adjuvant radiochemotherapy followed by chemotherapy with temozolomide, however prognosis is extremely poor. Assessment of the residual tumor after surgery and patient stratification into prognostic groups (i.e., by tumor volume) is currently hindered by the subjective evaluation of residual enhancement in medical images (magnetic resonance imaging [MRI]). Furthermore, objective evidence defining the optimal time to acquire the images is lacking. We analyzed 144 patients with glioblastoma, objectively quantified the enhancing residual tumor through computational image analysis and assessed the correlation with survival. Pathological enhancement thickness on post-surgical MRI correlated with survival (hazard ratio: 1.98, p < 0.001). The prognostic value of several imaging and clinical variables was analyzed individually and combined (radiomics AUC 0.71, p = 0.07; combined AUC 0.72, p < 0.001). Residual enhancement thickness and radiomics complemented clinical data for prognosis stratification in patients with glioblastoma. Significant results were only obtained for scans performed between 24 and 72 h after surgery, raising the possibility of confounding non-tumor enhancement in very early post-surgery MRI. Regarding the extent of resection, and in agreement with recent studies, the association between the measured tumor remnant and survival supports maximal safe resection whenever possible.

## Introduction

Glioblastoma is the most common primary brain tumor^1^. Although some treatments prolong survival, the prognosis of patients with glioblastoma is very poor, e.g. 2-year survival of 26.5%^2^. Standard of care therapy consists of maximum safe resection combined with adjuvant radiochemotherapy followed by chemotherapy with temozolomide. The extent of tumor resection is a relevant prognostic factor in this patient population^3–9^. Albert et al.established that the extent of resection should be evaluated with a magnetic resonance imaging (MRI) scan performed shortly after surgery, as inflammatory reparative changes can result in benign non-tumor contrast enhancement that can be misinterpreted as tumor remnants^10^. This is widely accepted in clinical practice, and most guidelines recommend performing early postoperative magnetic resonance (EPMR) to evaluate the extent of resection. Some guidelines suggest performing the EPMR scan within the first 72 h after surgery, while others are more restrictive and state with 48 h^11–13^. However, these recommendations rely on the experience and opinion of experts, rather than on formal clinical evidence. To the best of our knowledge, an in-depth evaluation of the optimal time range to perform the EPMR scan has not yet been described. An objective numerical assessment of the residual tumor enhancement to support this would allow identification of the optimal timing to evaluate the enhancement in the EPMR, and thus, the timing at which it is most informative for patient prognosis.

There is no consensus about the extent of resection to stratify prognostic groups. Some studies suggest an “all-or-nothing” approach^8^ in which complete resection should be achieved to improve patient survival. However, others suggest that it is possible to stratify several prognostic groups according to ranges of tumor resection^3–7,9,10,14,15^, namely < 75%, 75–95%, 95–100% and total resection.

An objective numerical evaluation of the residual tumor would allow analysis of a continuous range of values instead of subjective qualitative assessment, and help identify the best approach in regards to therapy and prognosis, while also being readily applicable and comparable between centers and studies. In this study, we focus on the enhancing residual tumor only. While there may be also non-enhancing residual tumor, a recent study has demonstrated an association between the post-operative enhancement volume and survival^16^. Therefore, it may be possible to identify additional prognosis value using more extensive image analysis of the enhancing tumor remnant in the post-surgical MRI. To this end, we focused on the post-surgical MRI and investigated radiomics and perfusion imaging features as an alternative evaluation to the extent of resection, which requires paired pre- and post-surgical MRI.

Image processing and radiomics extract quantifiable features from body tissues using characterization algorithms on the image spatial data. Radiomics is a lively topic of research that is shaping medical image assessment and interpretation, providing crucial information regarding tumor biology including glioblastomas^17,18^.

In this study, our goals were to develop a tool to facilitate the quantification of enhancing post-surgery residual tumor in patients with glioblastoma and analyze whether precise quantification of the area of contrast enhancement by image processing and radiomics could improve the patient´s prognostic evaluation. With this objective data, we also assessed the time range from surgery to the EPMR scan for an optimal association of residual tumor and overall survival (OS), to provide quantitative evidence for the optimal time range to perform the EPMR scan after surgery.

## Results

A total of 144 patients were included in the study (92 [64%] men, 52 [36%] women); median age of all patients was 59 years (range 20–77 years). Population demographics are described in Supplementary Table S1.

### Correlation of post-operative enhancement with survival

The enhancement thickness was calculated as the 3D distance transform of the segmented tumor, and mean and maximum values were determined. The mean and maximum enhancement thickness correlated with OS with a hazard ratio (HR) of 1.98 (95% CI 1.36–2.90, p < 0.001) and 1.11 (95% CI 1.05–1.17, p < 0.001), respectively (Table 1). Mean and maximum thickness also correlated significantly with progression-free survival (PFS) with HR of 2.01 (95% CI 1.35–3.12, p < 0.001) and 1.08 (95% CI 1.03–1.14, p = 0.004), respectively.

**Table 1:** Univariate Cox model of the enhancement thickness (mean and maximum) for overall survival, for the entire population and by subgroup according to time elapsed between surgery and the MRI.

Variable	Group	Distribution			Cox regression		
		n	Mean	SD	HR	95% CI	p-value
Mean thickness (mm)	All	144	1.18	0.44	1.98	1.36–2.90	< 0.001
	< 24 h	26	1.25	0.57	1.19	0.59–2.39	0.44
	24–48 h	51	1.18	0.39	3.00	1.24–7.24	0.03
	48–72 h	42	1.16	0.44	3.30	1.30–8.40	0.03
	≥ 72 h	25	1.21	0.51	2.40	0.74–7.72	0.16
Maximum thickness (mm)	All	144	7.16	3.27	1.11	1.05–1.17	< 0.001
	< 24 h	26	6.98	3.54	1.02	0.91–1.14	0.56
	24–48 h	51	7.41	3.23	1.21	1.08–1.36	0.005
	48–72 h	42	6.92	3.05	1.19	1.04–1.36	0.02
	≥ 72 h	25	7.16	3.81	1.08	0.96–1.21	0.21

P-values were adjusted for multiple test comparison.

*N* number of observations, *SD* standard deviation, *mm* millimetres, *h* hour, *HR* hazard ratio, *CI* confidence interval, *LR* likelihood ratio.

**Figure 1:**
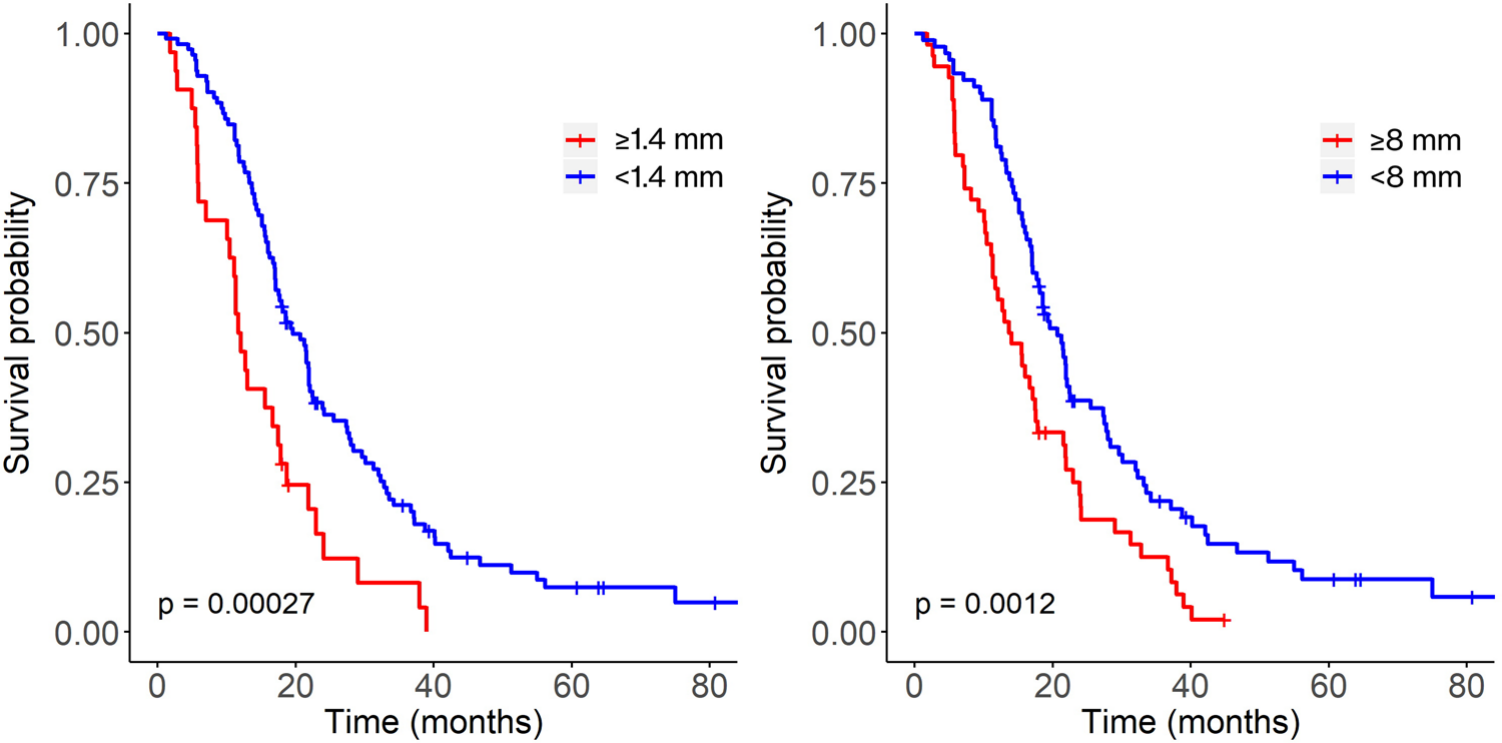
Kaplan–Meier survival curves for the enhancement thickness analysis, according to high and low mean (left) and maximum (right) thickness. Censored data are indicated with tick marks.

The survival rate of patients with a mean thickness of 1.4 mm or more was 47% after 12 months, whereas survival of patients with a mean thickness less than 1.4 mm was 78% (p < 0.001, Fig. 1, left panel). Similarly, the survival rate of patients with a maximum thickness of 8 mm or more was 55% after 12 months, whereas survival of patients with maximum thickness of less than 8 mm was 80% after 12 months (p = 0.0012, Fig. 1, right panel).

The volume of enhancement was also associated with OS, with an HR of 1.04 (95% CI 1.01–1.07, p = 0.009), however there was no significant association with PFS (HR 1.02, 95% CI 0.99–1.05, p = 0.19). A previously reported threshold of 12 mL^16^ resulted in unbalanced subpopulations: patients with enhancement volume greater than 12 mL (n = 10) had a survival rate of 20% at 12 months, while patients with volume less than 12 mL (n = 134) had a survival rate of 75% at 12 months (Supplementary Fig. S1). Despite the different survival rates, Kaplan–Meier analysis did not yield a significant difference (p = 0.1).

### Survival prediction for time between surgery and the EPMR scan

We analyzed the population in terms of the time elapsed between surgery and the EPMR scan according to the following groups: within 24 h (n = 26), from 24 up to 48 h (n = 51), from 48 up to 72 h (n = 42), and 72 h or more (n = 25). The mean and maximum thickness of enhancement were significantly associated with survival in patients with an EPMR scan performed between 24 and 72 h after surgery (p < 0.05, adjusted for multiple test comparisons, Table 1). There was no association between thickness and OS when the EPMR was performed within 24 h of surgery or more than 72 h after. In Supplementary Fig. S2, the HR and the confidence intervals of each subgroup are shown for comparison, and the Kaplan–Meier curves are shown in Supplementary Fig. S3.

### Prognostic value of perfusion sub-analysis

Dynamic susceptibility contrast (DSC) data were available for 113 patients (113/144, 78%). When the entire population was analyzed, patients with a relative cerebral blood volume in the 99th percentile (rCBV-99) above 8.26 had worse survival (p = 0.05, Supplementary Table S2), with a survival rate of 50% after 12 months, compared to a 75% rate in patients below this threshold (Supplementary Fig. S4). A maximum percentage of signal recovery (PSR) above 112% defined patients with lower survival (p < 0.001, Supplementary Table S2), with a 50% survival rate after 12 months, compared to an 80% rate for patients below the threshold (Supplementary Fig. S4).

We also analyzed the perfusion data in terms of the time between surgery and the EPMR scan, according to within the first 24 h (n = 22), between 24 and 48 h (n = 37), between 48 and 72 h (n = 33) and 72 h or later (n = 21). rCBV-99 predicted survival when the EPMR scan was performed 24 to 48 h post-surgery (p = 0.004 adjusted for multiple test comparisons, Supplementary Table S2); for patients with a rCBV-99 above 7.93, the survival rate was 20% after 12 months, compared to 70% for those below this threshold (Supplementary Fig. S4). The maximum PSR predicted survival when the EPMR scan was performed 24 to 72 h post-surgery (p < 0.001, adjusted for multiple test comparisons, Supplementary Table S2), with a survival rate of 20% after 12 months for patients above a 111% threshold and a rate of 80% for patients below this threshold (Supplementary Fig. S4). Neither the rCBV-99 nor PSR had prognostic value when the EPMR scan was performed within 24 h of surgery. When the scan was performed more than 72 h after surgery, the rCBV-99 showed significantly different survival rates (p = 0.011), albeit with unbalanced subgroups.

### Prognostic value of the radiomics signature

The population with measurable enhancement segmentation (129/144, 90%; see “Methods” for details) was analyzed to determine the prognosis of the clinical endpoint of OS ≥ 2 years (long; 36/129, 28%) or OS < 2 years (short; 93/129, 72%). The population was randomly split into training (92/129, 70%) and test (37/129, 30%) sets, with balanced distribution of survival in both sets (26/92, 28% of long survivors in the training; 10/37, 27% of long survivors in the test set) (Supplementary Table S3).

Twelve radiomics variables were selected in the training set using minimum-redundancy-maximum-relevance and stepwise regression (Supplementary Table S4). This radiomics signature predicted short and long survival groups with an AUC of 0.73 (0.60–0.86 p < 0.001) in the training set and an AUC of 0.71 (0.55–0.88 p = 0.01) in the test set. Receiver operating characteristic (ROC) curves and Kaplan–Meier curves of the training and test sets are presented in Supplementary Fig. S5.

### Combining quantitative imaging and clinical data for predicting survival

**Figure 2:**
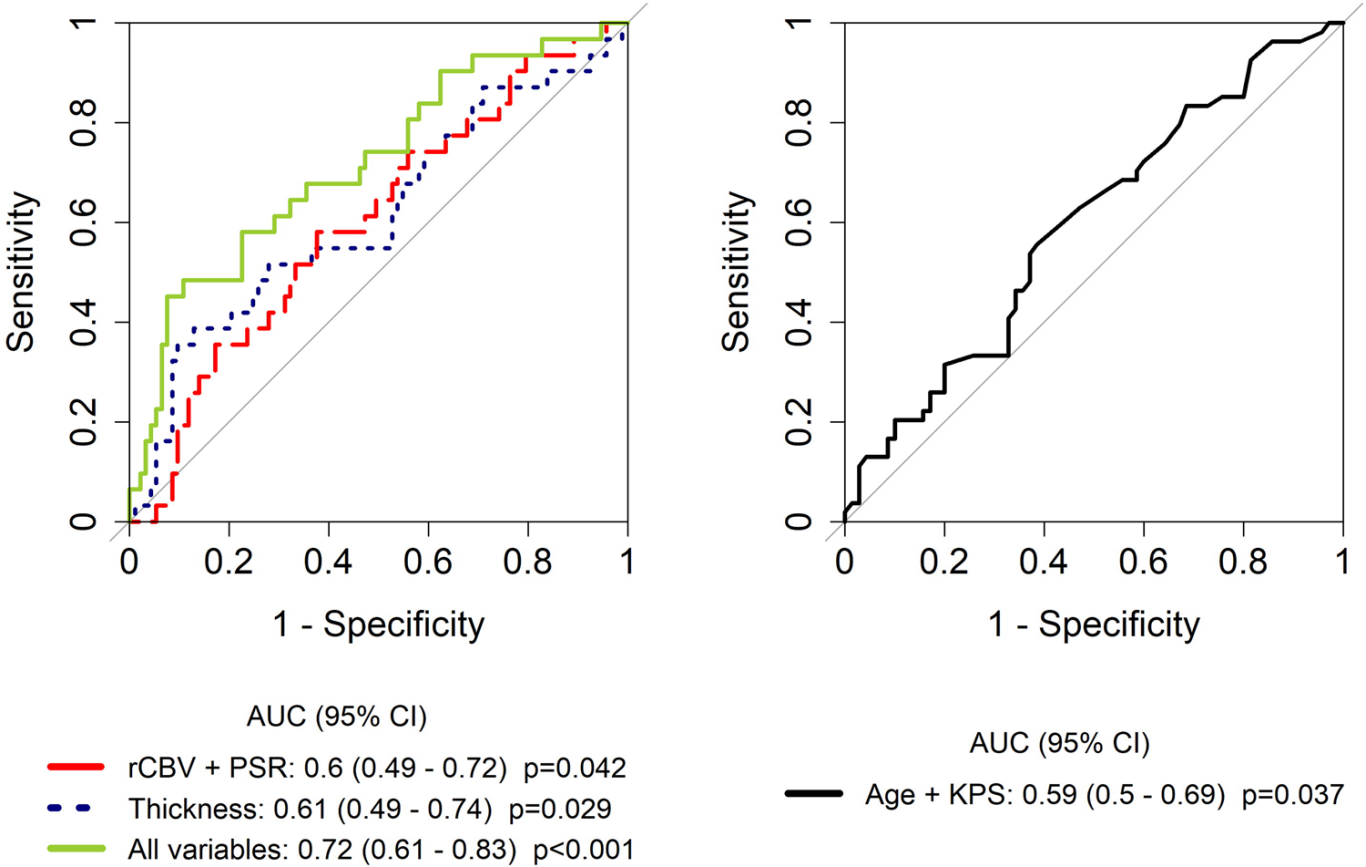
ROC of different prognostic models: perfusion variables (99th percentile cerebral blood volume [rCBV], maximum percentage of signal recovery [PSR]), mean thickness of enhancement and all variables (perfusion, thickness, age, Karnofsky performance status [KPS] and radiomics) together for predicting survival (left panel). The model with clinical variables age and KPS is shown in the right panel for clarity.

The multivariate logistic model including all imaging features (mean enhancement thickness, DSC, radiomics) and clinical data (age, postoperative KPS) yielded the highest prognostic capacity for predicting long and short survival (AUC 0.72, 95% CI 0.61–0.83, p < 0.001, Fig. 2). In the multivariate Cox model including all of these parameters, the mean thickness and age were retained as independent prognostic factors (Supplementary Table S5).

## Discussion

Radiological visual assessment of the remaining tumor after surgery is the standard of care in oncology clinical practice, and should be performed with an MRI rapidly after the surgical procedure. The extent of resection is a well-known prognostic factor in glioblastomas^4,5,8^, though inter-reader reproducibility is limited and hinders comparisons between centers and studies.

In this study, we identified a method to facilitate the quantification of the remaining tumor using a processing pipeline of multi-sequence MRI scans. By automatic registration and subtraction of T1w and T1wC images, it is possible to optimally isolate the enhancing areas from confounding post-operative changes. This method was also conceived by Ellingson et al.^16^, although they reported only the enhancement volume and did not analyze the influence of time to the EPMR, to measure post-surgery residual enhancing tumor. Moreover, we analyzed and internally validated a multivariate prognostic model including quantified residual tumor, perfusion, radiomics and clinical variables in an effort to improve the prognostic performance of residual tumor enhancement.

In our population, the quantification of the residual enhancement thickness after surgery shows a continuous association with OS and PFS (HR of 1.98 and 2.01 respectively, for mean enhancement thickness) that outperforms the subjective assessment of a dichotomous thin-or-thick tumor remnant^3^. Our findings also showed that the enhancement thickness had a stronger association with survival compared to the enhancement volume, suggesting the value of further investigation of this metric.

The combination of the enhancement thickness, perfusion, radiomics and clinical data into a predictive model showed slightly better performance (AUC 0.72) for distinguishing patients with short and long survival compared to thickness alone, compared to the univariate perfusion or the clinical models. When analyzed in separate groups, age and KPS yielded the lowest AUC (0.59), followed by rCBV and PSR (0.60), thickness (0.61) and radiomics (0.71). Therefore, in our population, radiomics variables performed better when applied in a dichotomous patient longer-or-shorter survival model, while the other variables added only marginal value. However, when correlating with specific patient survival, enhancement thickness alone demonstrated meaningful prognostic value, with higher mean thickness (as a continuous variable) associated with poorer survival (HR of 1.98 [95% CI 1.36–2.90, p < 0.001]). Both the enhancement thickness and the radiomics signature can be automatically calculated from the enhancement mask. Although further work is needed to facilitate the implementation of these assays in clinical practice, these results show the potential application of quantitative data from EPMR T1w and T1wC for supporting medical decisions.

We also explored the impact of the time between surgery and the MRI scan for quantifying the residual tumor. According to the National Comprehensive Cancer Network guideline, the early post-surgery MRI scan should be performed within the first 72 h after surgery^19^. Other guidelines are more restrictive and suggest that the scan be performed within the first 24 to 48 h after surgery^12,13,20^. The rationale for earlier imaging is that after 72 h post-surgery, inflammatory reparative changes result in benign non-tumor contrast enhancement that could be misinterpreted as tumor remnant^10^. Correspondingly, in our study we found no significant association between the measured enhancement and OS in the group of patients with EPMR scans performed more than 72 h after surgery.

Interestingly, we found no association between the enhancement thickness and survival when the MRI was acquired within the first 24 h after surgery. Although the sample size of this subpopulation was small (n = 26 patients), there was no visible trend towards the strong association found for the next time range. We hypothesize that immediately after surgery, intra-cavity transudation leads to non-tumor enhancement. Accordingly, Smets et al*.* showed that contrast transudation could appear in the very early postoperative MRI scans of glioblastomas^6^. Thereafter, the tissues may repair so that the enhancement corresponds to the real tumor remnants. Nevertheless, other possible explanations for this lack of association include the presence of surgical products that may affect MRIs performed soon after surgery.

We further explored whether features describing tissue vasculature from the DSC would differentiate the enhancement corresponding to post-surgery residual tumor and inflammation. Interestingly, 99th percentile rCBV and maximum PSR are indicators of bad prognosis when the residual tumor was assessed in an EPMR performed more than 24 h post-surgery. Accordingly, associations between recurrent tumor and higher rCBV values have been previously reported^21^. However, this correlation is strongest between 24 to 48 h but was not present for scans acquired before 24 h or after 72 h.

Taken together, these findings suggest that the enhancement thickness is representative of the remaining tumor when the MRI scan is acquired from 24 to 72 h after surgery, however there may be some confounders (i.e., non-tumor contrast enhancement) when the EPMR is acquired too early (during the first day after surgery) or too late (more than 4 days after surgery). Further studies are needed to better ascertain the importance of the number of days after surgery in measuring the remaining tumor tissue and elucidating the biological explanation behind such a phenomenon.

The prognostic value of the extent of resection is widely accepted by the scientific community. Nevertheless, there is no consensus about the threshold of the extent of resection that can influence survival. Some authors have suggested an “all-or-nothing” approach^8^. In this setting, no debulking surgery should be pursued when the tumor involves critical brain areas. Other studies however, suggest that a smaller extent of tumor resection could also positively impact patient survival^3–7^. In agreement with this, we found a correlation between residual tumor extent and patient survival. This supports maximal safe tumor resection with the knowledge that it could improve patient survival even in cases of partial tumor resection.

In our population, the radiomics analysis of the enhancement area including texture features, allows for identification of patients with long and short survival. As previously shown, radiomics features inform tumor heterogeneity and are indicators of tumor aggressiveness in glioblastoma^22–24^. We have explored several other variables to evaluate their clinical utility. While all quantitative imaging data (radiomics, thickness, DSC) provide relevant information that complements clinical data (age and KPS), we found the enhancing tumor thickness and radiomics to have high prognostic value and only T1w and T1wC are required to calculate them, thus simplifying the implementation.

This study has a number of potential limitations. Firstly, as a retrospective study, isocitrate dehydrogenase (*IDH)* mutation status data were available for 54 patients, only one of whom (2%) had an *IDH* mutation. This low incidence rate may be explained by the relatively old age of our population, as *IDH*-mutant glioblastoma has been reported to correlate with younger age^25^. In addition, only glioblastomas treated with maximal safe resection were included in our study. Newly diagnosed *IDH*-mutant glioblastoma tend to present with a more diffuse infiltrative pattern than *IDH*-wildtype glioblastoma, likely leading to a biopsy instead of a maximal safe resection, and *IDH*-mutant glioblastomas progressing after a previous known *IDH*-mutant low-grade astrocytoma^26^ were also not included in our study. Therefore, there may be confounders in patients for whom *IDH* status is missing, although as the rate of *IDH* mutant in glioblastoma is 10% in the general population^27^ compared to approximately 2% in our population, we believe this did not significantly affect our results.

Secondly, in this analysis we focus on the post-surgical residual enhancing tumor and certain drugs may be administered perioperatively that can affect the enhancement on post-surgery T1wC scans (i.e., glucocorticoids or antiangiogenic drugs such as bevacizumab). We confirmed that bevacizumab was not administered prior to EPMR in any patients. Glucocorticoids might have been administered in cases with extensive edema, which could have influenced the characteristics of post-contrast MRI. Nevertheless, glucocorticoids are avoided as much as possible in our hospital to prevent this drug from affecting the histological interpretation of biopsy samples.

Thirdly, given the observational retrospective nature of this study, standardization of all the image acquisition parameters was not possible. Therefore, all images were homogenously normalized in intensity and resampled to minimize variability. Additionally, DSC scans without contrast preload and acquisition at 1.5 T may result in leakage effects, hence leakage correction methods were applied to avoid confounders. Additionally, the method used requires input (semi-automatic segmentation) from an experienced radiologist who supervises the enhancement mask. This might induce some segmentation variability. However, the thickness is calculated as the mean value of the distance transform, and thus it is possible that an average value smooths out small differences in segmentations, making it more robust. Lastly, we performed an internal validation, though applying the prognostic models to an external dataset would confirm its performance.

It is noteworthy to mention that there may still be non-enhancing residual tumor, which is out of the scope of the current analysis. Therefore, as future work, combining other sequences such as T2w and FLAIR (Fluid-Attenuated Inversion Recovery) may be useful to delineate additional areas of residual tumor with a potential role in patient prognosis^28,29^, although at the expense of a more complex pipeline.

Additionally, novel MRI techniques using chemical exchange saturation transfer (CEST) imaging have been reported to correlate with response to treatment and survival. With CEST MRI, pulses off the water resonance frequency are absorbed by proteins that then transfer the energy to water through chemical exchange, lowering the signal. Derived signal maps such as amide proton transfer (APT) and nuclear Overhauser effect (NOE) are both reported to correlate with survival in gliomas and glioblastomas^30–32^, even in a sub-cohort of *IDH*-wildtype patients. While the number of patients evaluated in these studies is small, they showed similar HRs as those reported in our study. This highlights the potential of CEST MRI and its radiomics analysis, and merits further research. In combination with the relevant variables proposed in this study, a thorough imaging signature could provide major insight into patient response.

In conclusion, in patients with glioblastoma multiforme, objective quantification of the area of enhancement in the tumor bed after surgery, in an EPMR scan performed between 24 and 72 h after surgery provides relevant information on the remaining tumor and relevant prognostic information. The MRI processing pipeline defined in this study is an easy and rapid method for a more accurate evaluation of the post-surgery residual tumor, that could be implemented in routine clinical practice.

## Methods

### Ethical approval

The Research Ethics Committee of the Hospital Universitari de Bellvitge revised and approved this study, in compliance with applicable regulatory requirements and the International Conference on Harmonization Guidelines on Good Clinical Practice (ICH GCP). For this retrospective study, informed consent was waived by the Research Ethics Committee of the Hospital Universitari de Bellvitge.

### Study population and design

We evaluated data from 160 consecutive patients with primary glioblastoma multiforme who underwent maximum safe resection surgery between February 2009 and December 2017 at the Hospital Universitari de Bellvitge, Spain, and who had an early post-surgery MRI scan (i.e., within the first week after surgery). Data on isocitrate dehydrogenase (*IDH*) 1/2 mutation status were available in 54 out of 160 patients (33%)^33^. Only one patient (1/54, 2%) had an identified *IDH* mutation. To be eligible for the analysis, patients had to have been treated with adjuvant radiotherapy plus concomitant and post-radiotherapy temozolomide (i.e., Stupp protocol)^2^ (144/160, 90%).

### MRI scan protocol and image pre-processing

The earliest post-surgery MRI scan per patient was collected, including T1-weighted before (T1w) and after intravenous contrast administration (T1wC), and dynamic susceptibility contrast (DSC) images. Approximately half of the MRI scans were acquired with a 1.5 T MR magnet Intera (Philips Healthcare, Best, The Netherlands) and half with a 1.5 T MR magnet Achieva (Philips Healthcare, Best, The Netherlands) with the following parameters for T1w: spin-echo; TE 15 ms; TR 540 ms; flip angle 90°; matrix size 256 × 256 mm^2^; slice thickness 5 mm.

A single MRI scan was performed with a 3 T MR unit (Achieva; Philips Healthcare, Best, The Netherlands), gradient-echo; TE 4 ms; TR 9 ms; flip angle 8º; matrix size 672 × 672 mm^2^; slice thickness 1 mm.

Regarding DSC, all acquisitions were gradient-echo without bolus preload; a total of 10 baseline points were collected before the pass of contrast with the following protocol: in the 1.5 T Intera MR unit (scans from 2009 to 2015) PRESTO sequence; TE 25 ms; TR 17 ms; flip angle 7°; matrix size 128 × 128 mm^2^; slice thickness 3.5 mm; dynamic acquisitions 40. In the 1.5 T Achieva MR unit (from 2015 to 2917) TE 40 ms; TR 1522–1642 ms; flip angle 75º; matrix size 128 × 128 mm^2^; slice thickness, 4–5 mm; dynamic acquisitions 60.

A single MRI scan was performed with a 3 T MR unit (Achieva; Philips Healthcare, Best, The Netherlands), echo planar sequence; TE 40 ms; TR 1618 ms; flip angle 75º; matrix size 128 × 128 mm^2^; slice thickness 4 mm; dynamic acquisitions 40.

The pre-processing of images for homogenization included N4 bias field correction^34^ and intensity normalization with Nyul’s method as adapted by Shah^35,36^.

### Enhancement mask definition and thickness quantification

The T1w and T1wC images with different slice spacing (1/144 = 1% of the scans) were resampled to 5 mm for consistency and robustness. The T1w was registered to the T1wC with the rigid transformation tool of 3DSlicer v4.10^37^. The T1w signal intensity was normalized to that of the T1Cw^28^. A 3D enhancement map was obtained by T1w-T1wC subtraction. The area of enhancement within and around the tumor bed was delineated by an experienced radiologist (P.N.B), blinded to the clinical outcome, using the 3DSlicer semi-automatic delineation tools (thresholding and morphological operations).

**Figure 3:**
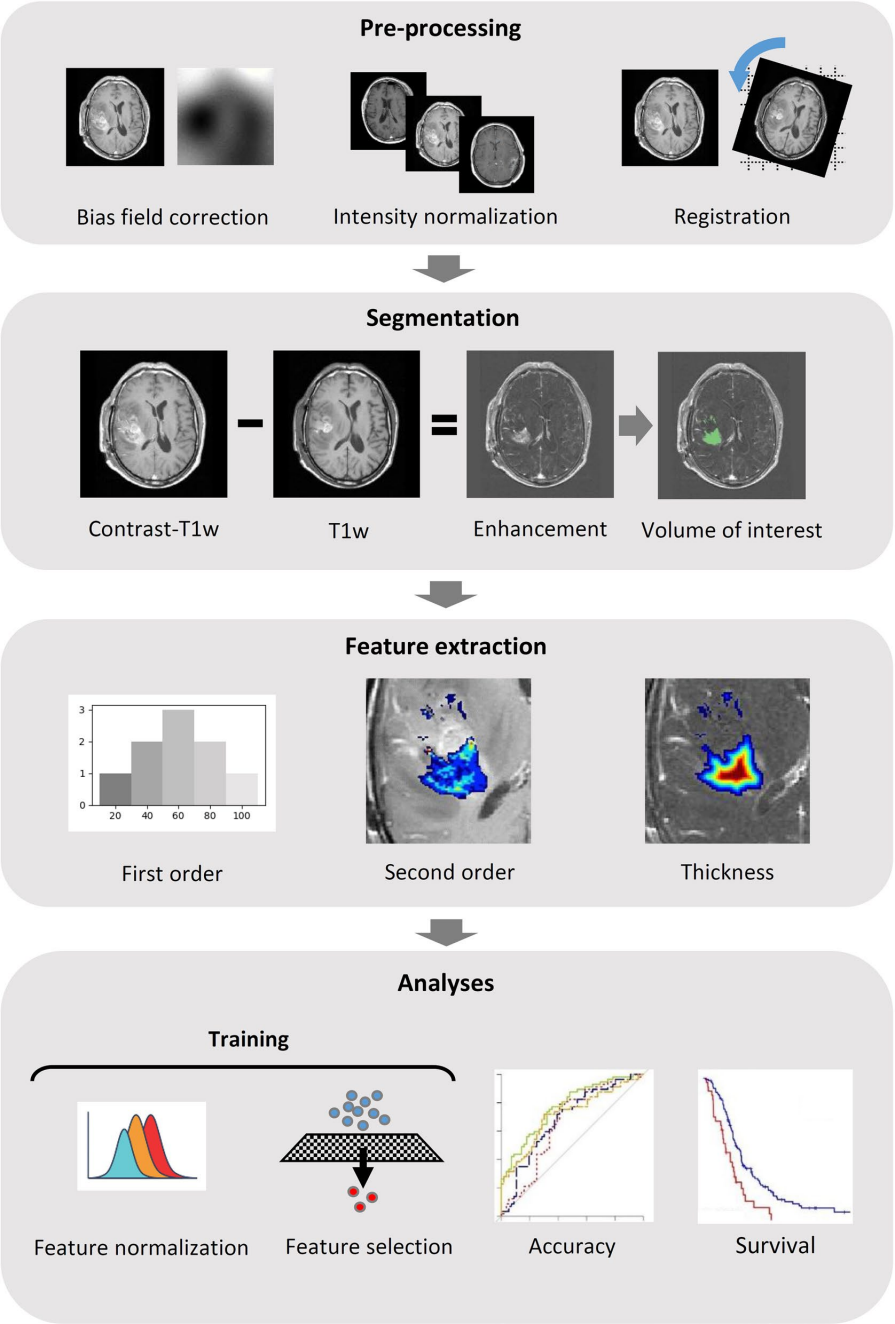
Pipeline of the applied methods. Registration is performed with the contrast-T1w scan as the reference image and the radiomics features are extracted from the contrast-T1w. The thickness is calculated from the 3D distance transform of the volume of interest, depicted as a colormap.

The 3D distance transform of the volume of interest (VOI) was then calculated. The distance transform provides the Euclidean distance in millimeters of every voxel within the VOI to its nearest VOI boundary^38^. We calculated the mean and the maximum values of the 3D distance transform as measures of the enhancement mask thickness for each patient (Fig. 3), as well as the total volume of the VOI.

### Perfusion MRI sub-analysis

The DSC temporal volumes were processed with the DSCMRIAnalysis module of 3DSlicer^37,39^, the curves were leakage-corrected with the Boxerman–Schmainda–Weiskoff method^40^ and the rCBV were obtained. The DSC volumes were then registered to the T1wC images for co-alignment with the enhancement masks. The temporal curves of the DSC were first analyzed to remove noisy and low-signal curves from the mask. For this purpose only, the curves were low-pass filtered, normalized between − 1 and 0, and then those that reflected the bolus arrival^41,42^ were selected by resemblance to a Gaussian distribution (Supplementary Fig. S6). Once the DSC curves had been filtered, the original non-normalized DSC values were analyzed.

### Relative cerebral blood volume

An ROI within the white matter contralateral to the tumor was delineated in each MRI and the relative cerebral blood volume (rCBV) map was normalized to this ROI. To retrieve the tumor hot-spot, we calculated the 99th percentile of the rCBV (rCBV-99) and the maximum percentage of signal recovery (PSR) from the enhancement mask selected curves. The rCBV-99 and not the maximum was calculated to avoid extreme outlier values of the rCBV. The PSR was calculated as described by Cha et al*.*^42^.

### Radiomics feature extraction and robustness

Radiomics extraction was performed with Pyradiomics v2.1.2 for Python^43^, with image resampling of 1 mm isotropic voxels and fixed binarization to 10 levels of bin-width, as has been suggested to maximize radiomics reproducibility^44–46^. Ninety-four radiomics features were extracted from the enhancement mask applied to the T1wC images, including first-order (n = 19) and second-order from texture Grey-Level Co-occurrence Matrix (n = 24), Grey-Level Run-Length Matrix (n = 16), Grey-Level Size-Zone Matrix (n = 16), Neighboring-Grey-Tone Difference Matrix (n = 5) and Grey-Level Dependence Matrix (n = 14) (Supplementary Table S6). Feature description and compliance with image biomarker standardization initiative guidelines is publicly available in the package documentation^43^. We also studied the variability of radiomics when changing the extraction parameters voxel size and bin width, and when the most robust features (i.e., < 20% coefficient of variation) were selected for further analysis (54 out of 94 [57%] radiomics features, see Supplementary Methods).

### Radiomics feature selection and analysis

Patients with measurable enhancement segmentation (129/144, 90%) were eligible for this analysis. For clarification, these patients may present gross total resection, however benign reactive enhancement has been reported by some authors up to 72 h after surgery^3,6,16,47^. The patients were split into ‘training’ (70%) and ‘test’ (30%) sets for internal validation, with balanced survival distribution in both sets. Patient characteristics of the sets are presented in Supplementary Table S3. The radiomics features were normalized according to the mean value and standard deviation of the training set. From the 54 robust features of the previous section, further selection was performed in the training set by minimum-redundancy–maximum-relevance^48,49^ plus stepwise regression. The logistic regression model with the selected variables was then internally validated in the test set.

### Statistical analysis

Log-rank analysis on Kaplan–Meier data and Cox proportional hazards regression models were used to evaluate the association of the analyzed variables with survival.

For dichotomized survival analysis, the population was split according to lower or higher than 2-year overall survival (< 2 years, ≥ 2 years respectively, Supplementary Table S1). Censored patients with a survival shorter than the defined endpoint were excluded. These survival groups were defined for logistic regression models and to obtain the area under the curve (AUC) of the receiver operating characteristic (ROC) curve. A threshold for each independent variable was calculated from the regression models from the maximum sum of sensitivity and specificity (Youden’s index). The postoperative Karnofsky performance status (KPS) was dichotomized as < 90 and ≥ 90.

To assess how the time from surgery to the EPMR scan affects the residual tumor quantification, sub-analyses of survival prediction were performed based on the time elapsed between surgery and the EPMR scan. The patients were split into four subpopulations according to the time between surgery and the MRI scan (< 24, 24 to < 48, 48 to < 72 and ≥ 72 h). Multiple comparison tests were adjusted with the Benjamini–Hochberg method, and p-values were considered statistically significant below 0.05.

The thickness analysis was done in-house with Matlab R2015a (Mathworks). Radiomics selection and statistical analysis were performed with R Statistical Software v3.5.1^50^.

## Supplementary Information

Supplementary Information

## Abbreviations

AUC: Area under the curve
CI: Confidence interval
DSC: Dynamic susceptibility contrast
EPMR: Early post-operative magnetic resonance
HR: Hazard ratio
IDH: Isocitrate dehydrogenase
KPS: Karnofsky performance status
LR: Likelihood ratio
PSR: Percentage of signal recovery
rCBV: Relative cerebral blood volume
ROI: Region of interest
OS: Overall survival
VOI: Volume of interest
W: Weighted

## References

[1] Ostrom QT (2018). CBTRUS statistical report: Primary brain and other central nervous system tumors diagnosed in the United States in 2011–2015. Neuro Oncol..

[2] Stupp R (2005). Radiotherapy plus concomitant and adjuvant temozolomide for glioblastoma. N. Engl. J. Med..

[3] Majos C (2016). Early post-operative magnetic resonance imaging in glioblastoma: Correlation among radiological findings and overall survival in 60 patients. Eur. Radiol..

[4] Kuhnt D (2011). Correlation of the extent of tumor volume resection and patient survival in surgery of glioblastoma multiforme with high-field intraoperative MRI guidance. Neuro Oncol..

[5] Bloch O (2012). Impact of extent of resection for recurrent glioblastoma on overall survival: Clinical article. J. Neurosurg..

[6] Smets T, Lawson TM, Grandin C, Jankovski A, Raftopoulos C (2013). Immediate post-operative MRI suggestive of the site and timing of glioblastoma recurrence after gross total resection: A retrospective longitudinal preliminary study. Eur. Radiol..

[7] Krivoshapkin AL (2019). Automated volumetric analysis of postoperative magnetic resonance imaging predicts survival in patients with glioblastoma. World Neurosurg..

[8] Lacroix M (2001). A multivariate analysis of 416 patients with glioblastoma multiforme: Prognosis, extent of resection, and survival. J. Neurosurg..

[9] Ellingson BM (2018). Validation of postoperative residual contrast-enhancing tumor volume as an independent prognostic factor for overall survival in newly diagnosed glioblastoma. Neuro Oncol..

[10] Albert FK, Forsting M, Sartor K, Adams HP, Kunze S (1994). Early postoperative magnetic resonance imaging after resection of malignant glioma: Objective evaluation of residual tumor and its influence on regrowth and prognosis. Neurosurgery.

[11] Stupp R (2014). High-grade glioma: ESMO clinical practice guidelines for diagnosis, treatment and follow-up. Ann. Oncol.

[12] Wen PY (2010). Updated response assessment criteria for high-grade gliomas: Response assessment in neuro-oncology working group. J. Clin. Oncol..

[13] Martinez-Garcia M (2018). SEOM clinical guidelines for diagnosis and treatment of glioblastoma (2017). Clin. Transl. Oncol..

[14] Allahdini F, Amirjamshidi A, Reza-Zarei M, Abdollahi M (2010). Evaluating the prognostic factors effective on the outcome of patients with glioblastoma multiformis: Does maximal resection of the tumor lengthen the median survival?. World Neurosurg..

[15] Sanai N, Polley MY, McDermott MW, Parsa AT, Berger MS (2011). An extent of resection threshold for newly diagnosed glioblastomas. J. Neurosurg..

[16] Ellingson BM (2018). Validation of postoperative residual contrast-enhancing tumor volume as an independent prognostic factor for overall survival in newly diagnosed glioblastoma. Neuro-Oncology.

[17] Aerts HJ (2014). Decoding tumour phenotype by noninvasive imaging using a quantitative radiomics approach. Nat. Commun..

[18] Lambin P (2017). Radiomics: The bridge between medical imaging and personalized medicine. Nat. Rev. Clin. Oncol..

[19] National Comprehensive Cancer Network. Central Nervous System Cancers (Version 1.2019). NCCN Clin Pract Guidel Oncol 2019. https://www.nccn.org/professionals/physician_gls/ (accessed April 22, 2019).

[20] Stupp R (2014). High-grade glioma: ESMO clinical practice guidelines for diagnosis, treatment and follow-up. Ann. Oncol..

[21] Bisdas S (2009). Cerebral blood volume measurements by perfusion-weighted MR imaging in gliomas: Ready for prime time in predicting short-term outcome and recurrent disease?. AJNR Am. J. Neuroradiol..

[22] Li Q (2017). A fully-automatic multiparametric radiomics model: towards reproducible and prognostic imaging signature for prediction of overall survival in glioblastoma multiforme. Sci. Rep..

[23] Su C (2019). Radiomics based on multicontrast MRI can precisely differentiate among glioma subtypes and predict tumour-proliferative behaviour. Eur. Radiol..

[24] Ditmer A (2018). Diagnostic accuracy of MRI texture analysis for grading gliomas. J. Neurooncol..

[25] Robinson C, Kleinschmidt-DeMasters BK (2017). IDH1-mutation in diffuse gliomas in persons age 55 years and over. J. Neuropathol. Exp. Neurol..

[26] Ohgaki H, Kleihues P (2013). The definition of primary and secondary glioblastoma. Clin. Cancer Res..

[27] Louis DN (2016). The 2016 world health organization classification of tumors of the central nervous system: A summary. Acta Neuropathol..

[28] Rathore S (2018). Radiomic MRI signature reveals three distinct subtypes of glioblastoma with different clinical and molecular characteristics, offering prognostic value beyond IDH1. Sci. Rep..

[29] Porz N (2014). Multi-modal glioblastoma segmentation: Man versus machine. PLoS ONE.

[30] Meissner JE (2019). Early response assessment of glioma patients to definitive chemoradiotherapy using chemical exchange saturation transfer imaging at 7 T. J. Magn. Reson. Imaging.

[31] Paech D (2019). Relaxation-compensated amide proton transfer (APT) MRI signal intensity is associated with survival and progression in high-grade glioma patients. Eur. Radiol..

[32] Mehrabian H, Myrehaug S, Soliman H, Sahgal A, Stanisz GJ (2018). Evaluation of glioblastoma response to therapy with chemical exchange saturation transfer. Int. J. Radiat. Oncol. Biol. Phys..

[33] Yan H (2009). IDH1 and IDH2 mutations in gliomas. N. Engl. J. Med..

[34] Tustison NJ (2010). N4ITK: improved N3 bias correction. IEEE Trans. Med. Imaging.

[35] Nyul LG, Udupa JK (1999). On standardizing the MR image intensity scale. Magn. Reson. Med..

[36] Shah M (2011). Evaluating intensity normalization on MRIs of human brain with multiple sclerosis. Med. Image Anal..

[37] Fedorov A (2012). 3D slicer as an image computing platform for the quantitative imaging network. Magn. Reson. Imaging.

[38] Grevera, G.J. in Deformable* Models* Ch. Chapter 2, 33–60 (2007).

[39] Schmainda KM (2018). Multisite concordance of DSC-MRI analysis for brain tumors: Results of a national cancer institute quantitative imaging network collaborative project. AJNR Am. J. Neuroradiol..

[40] Boxerman JL, Schmainda KM, Weisskoff RM (2006). Relative cerebral blood volume maps corrected for contrast agent extravasation significantly correlate with glioma tumor grade, whereas uncorrected maps do not. Am. J. Neuroradiol..

[41] Lupo JM, Cha S, Chang SM, Nelson SJ (2005). Dynamic susceptibility-weighted perfusion imaging of high-grade gliomas: Characterization of spatial heterogeneity. AJNR Am. J. Neuroradiol..

[42] Cha S (2007). Differentiation of glioblastoma multiforme and single brain metastasis by peak height and percentage of signal intensity recovery derived from dynamic susceptibility-weighted contrast-enhanced perfusion MR imaging. AJNR Am. J. Neuroradiol..

[43] van Griethuysen JJM (2017). Computational radiomics system to decode the radiographic phenotype. Cancer Res..

[44] Molina D (2017). Lack of robustness of textural measures obtained from 3D brain tumor MRIs impose a need for standardization. PLoS ONE.

[45] Goya-Outi J (2018). Computation of reliable textural indices from multimodal brain MRI: Suggestions based on a study of patients with diffuse intrinsic pontine glioma. Phys. Med. Biol..

[46] Duron L (2019). Gray-level discretization impacts reproducible MRI radiomics texture features. PLoS ONE.

[47] Bette S (2016). Patterns and time dependence of unspecific enhancement in postoperative magnetic resonance imaging after glioblastoma resection. World Neurosurg..

[48] De Jay N (2013). mRMRe: An R package for parallelized mRMR ensemble feature selection. Bioinformatics.

[49] Parmar C (2015). Radiomic machine-learning classifiers for prognostic biomarkers of head and neck cancer. Front. Oncol..

[50] Core Team R (2013). R: A Language and Environment for Statistical Computing.

