# Supplementary material for: Precise enhancement quantification in post-operative MRI as an indicator of residual tumor impact is associated with survival in patients with glioblastoma
Source: Sci Rep. 2021 Jan 12;11:695. doi: 10.1038/s41598-020-79829-3 (PMC7804103; doi:10.1038/s41598-020-79829-3)
Supplement: Supplementary file 1 — Supplementary Information [file 41598_2020_79829_MOESM1_ESM.docx]

**Precise enhancement quantification in post-operative MRI as an indicator of residual tumor impact is associated with survival in patients with glioblastoma.**

Alonso Garcia-Ruiz^1,+^, Pablo Naval-Baudin^2,+^, Marta Ligero^1^, Albert Pons-Escoda^2,3^, Jordi Bruna^3,4^, Gerard Plans^3,5^, Nahum Calvo^2^, Monica Cos^2^, Carles Majós^2,3,++^, Raquel Perez-Lopez^1,6,++^*

^1^ Radiomics Group, Vall d’Hebron Institute of Oncology (VHIO), Barcelona, Spain.

^2^ Department of Radiology, Institut de Diagnòstic per la Imatge (IDI), Bellvitge University Hospital, Barcelona, Spain.

^3^ Neuro-Oncology Unit, Institut d'Investigació Biomèdica de Bellvitge (IDIBELL), Barcelona, Spain.

^4^ Department of Neurology, Bellvitge University Hospital, Barcelona, Spain.

^5^ Department of Neurosurgery, Bellvitge University Hospital, Barcelona, Spain.

^6^ Department of Radiology, Vall d’Hebron University Hospital, Barcelona, Spain.

**^+^ A.G.R. and P.N.B. are joint first authors.**

**^++^ C.M. and R.P.L. are joint senior authors.**

*** Corresponding author:**

Raquel Perez-Lopez

Radiomics Group, Vall d’Hebron Institute of Oncology

117 Natzaret, 08035, Barcelona (Spain)

Telephone: +34 93 2543450; email: rperez@vhio.net

Supplementary Material

**Supplementary Figure S1. Kaplan-Meier survival curves for the enhancing residual tumor volume. A previously reported volume of 12 ml [1] was applied as a threshold to split the population, resulting in unbalanced subgroups (>12ml n=10; <12ml n=134) and no significant difference (p=0.1). Censored data is indicated with tick marks.**


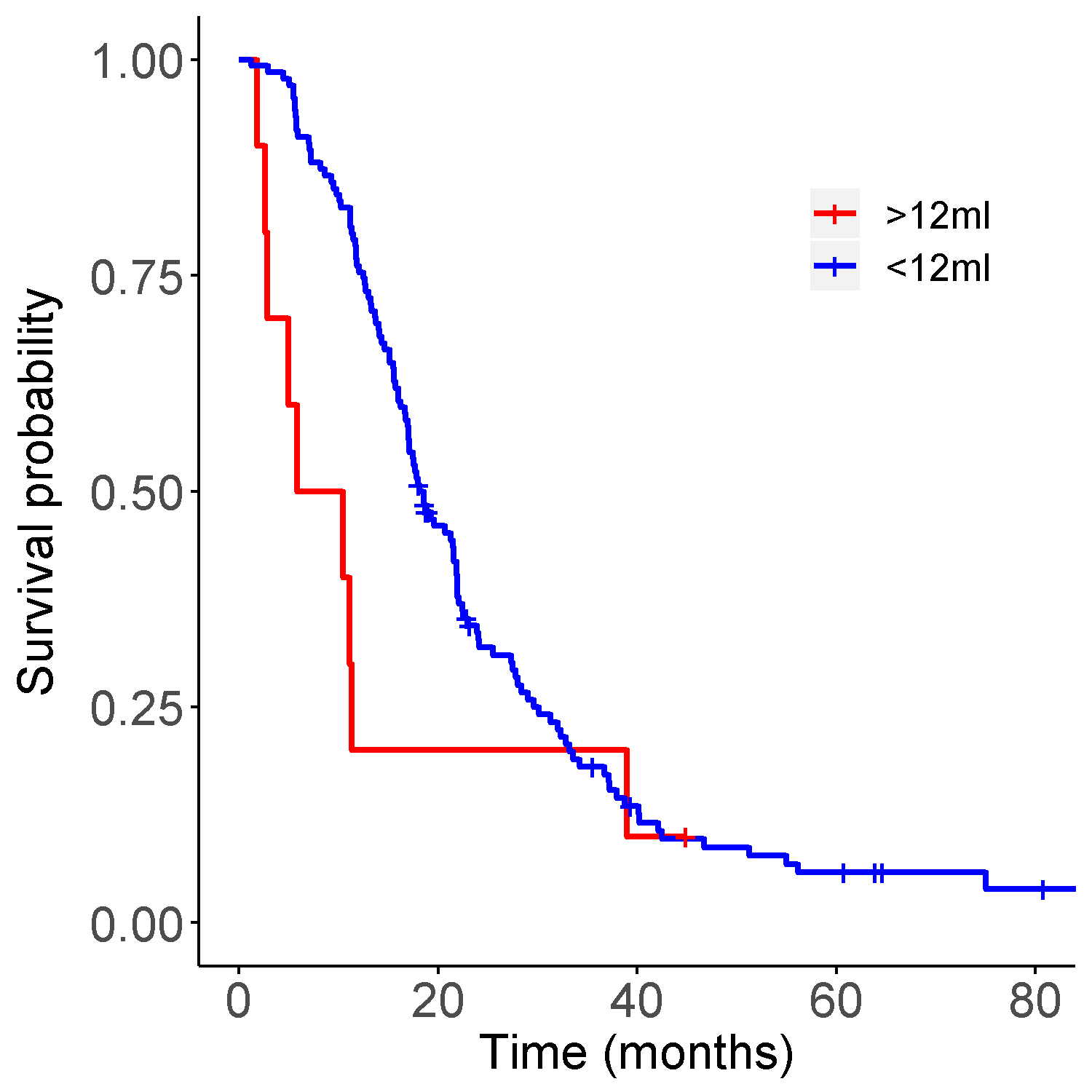


**Supplementary Figure S2. Comparison of the prognosis models by the enhancement thickness. The hazard ratios of the univariate Cox model for every group according to the time from surgery to the early MRI. The vertical lines indicate the 95% confidence interval. The dashed horizontal line indicates model relevance (hazard ratio above or below 1).**


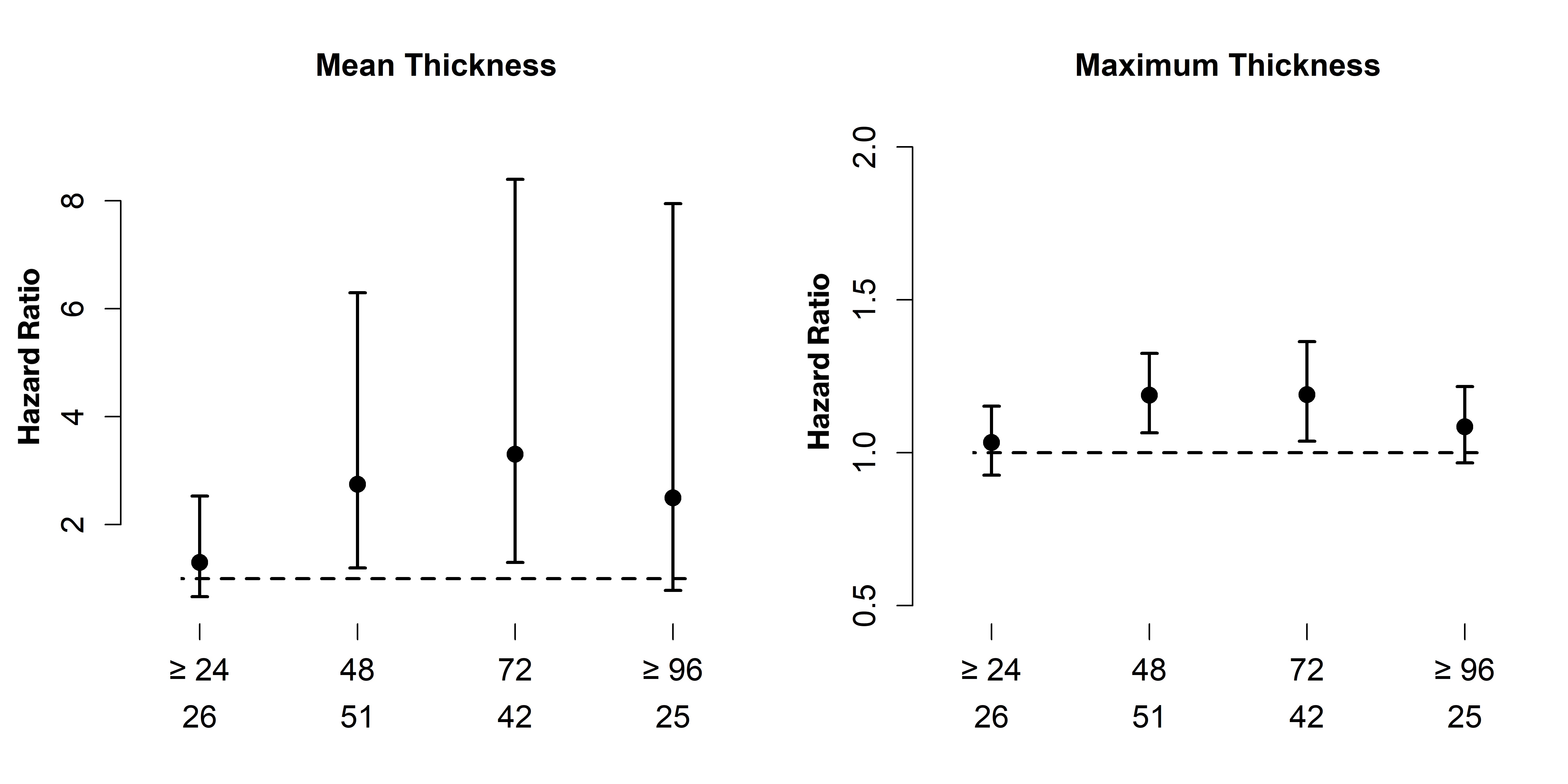


**Supplementary Figure S3. Log-rank analysis on Kaplan-Meier curves for mean and maximum thickness over different time ranges from the surgery to the MRI scan acquisition. From top to bottom: early post-operative magnetic resonance (EPMR) performed within less than 24 hours from surgery, EPMR between 24-48 hours from surgery, EPMR between 48-72 hours from surgery and ≥72 hours. Censored data is indicated with tick marks. P-values were adjusted for multiple test comparison.**


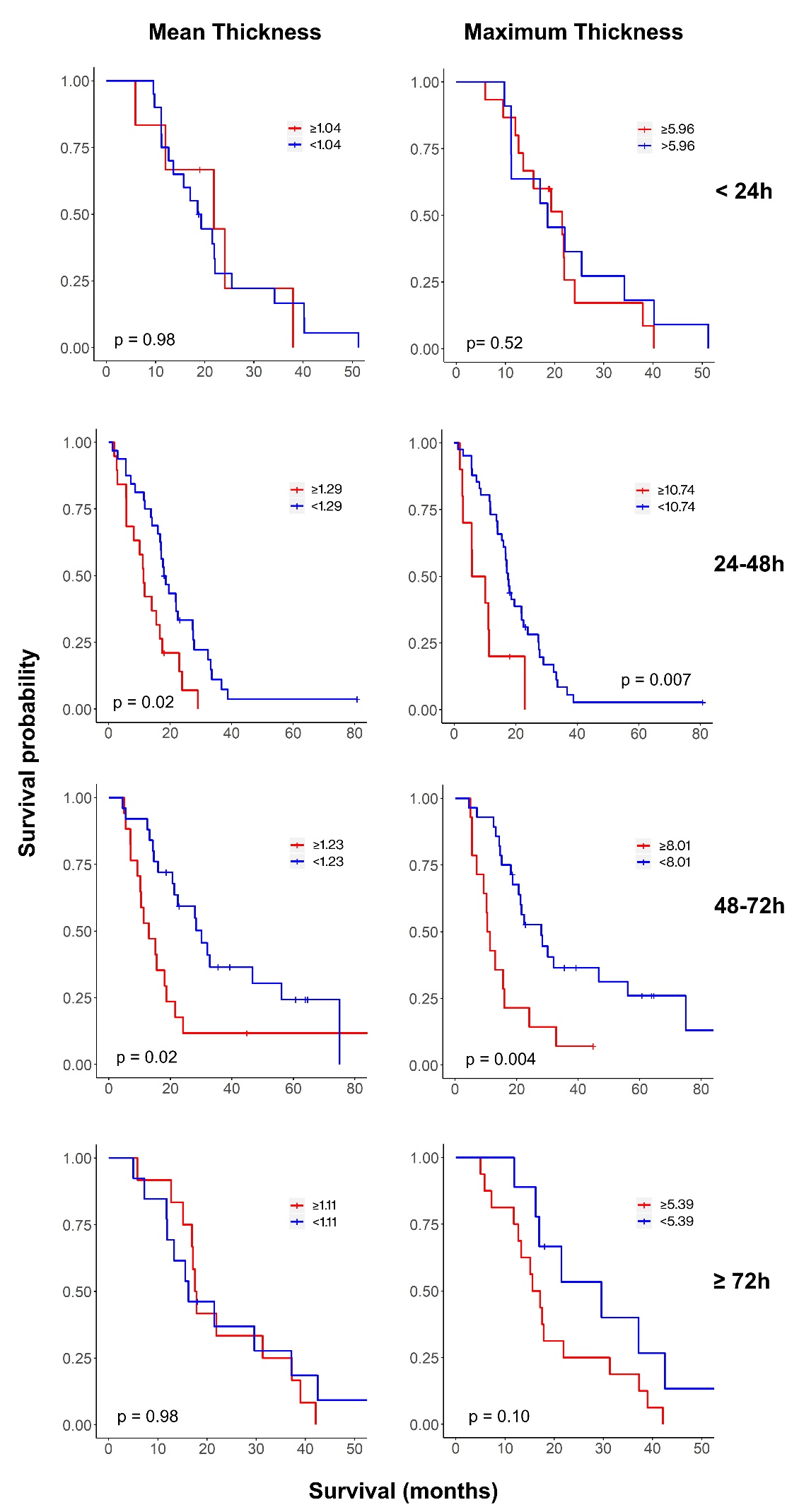


**Supplementary Figure S4. Log-rank analysis on Kaplan-Meier curves for perfusion variables (relative cerebral blood volume [rCBV] and percentage of signal recovery [PSR]). The 99th percentile rCBV and PSR were dichotomized by Youden’s index (Supplementary Table S2). From top to bottom: all the population, early post-operative magnetic resonance (EPMR) performed within less than 24 hours from surgery, EPMR between 24-48 hours from surgery, EPMR between 48-72 hours from surgery and ≥72 hours. Censored data is indicated with tick marks. P-values were adjusted for multiple test comparison.**


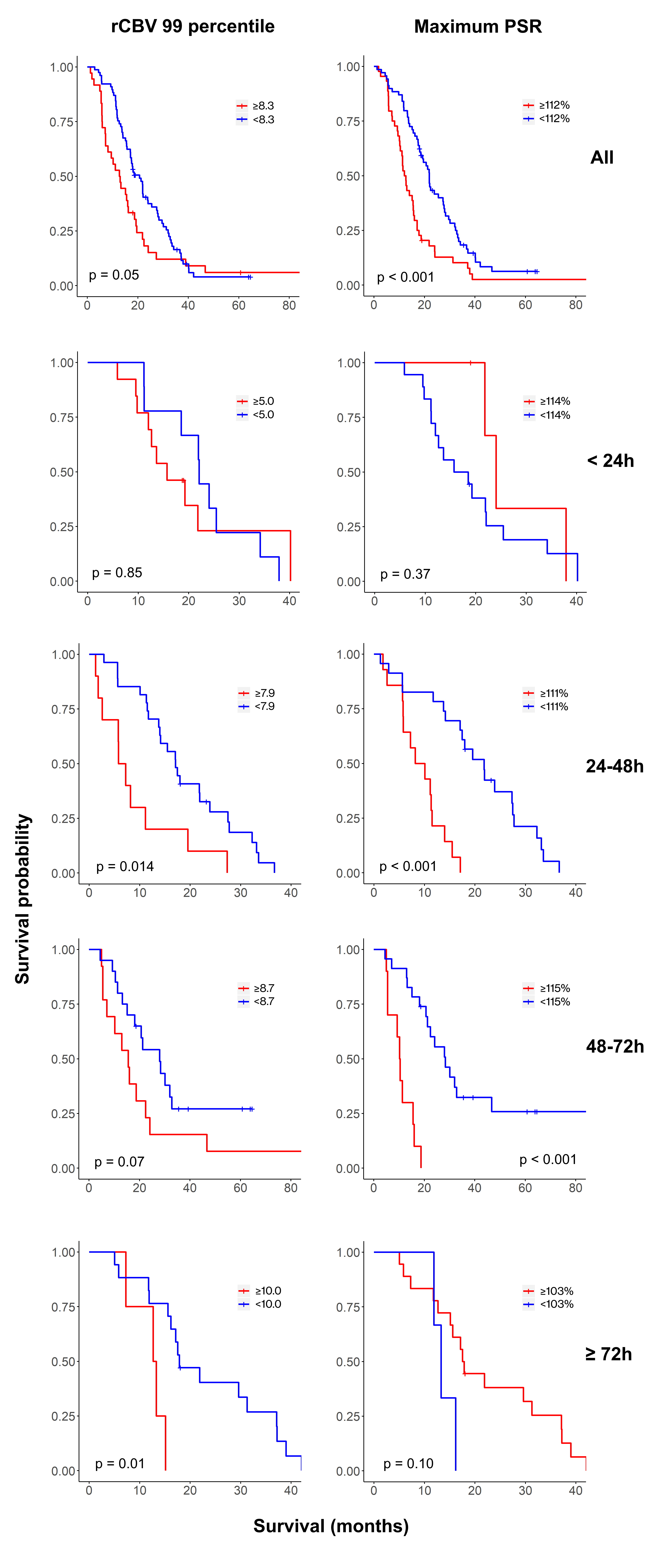


**Supplementary Figure S5. ROC curves of the logistic model of the radiomics variables applied in the training (top left) and test (top right) sets. Log-rank analysis on Kaplan-Meier curves; patients with a high radiomics score (i.e. above 0.5, defined by the ROC curve) showed an improved survival compared to those with low radiomics score (i.e. below 0.5) in the training (lower left) and test (lower right) sets, p=0.0011 and P=0.007 respectively.**


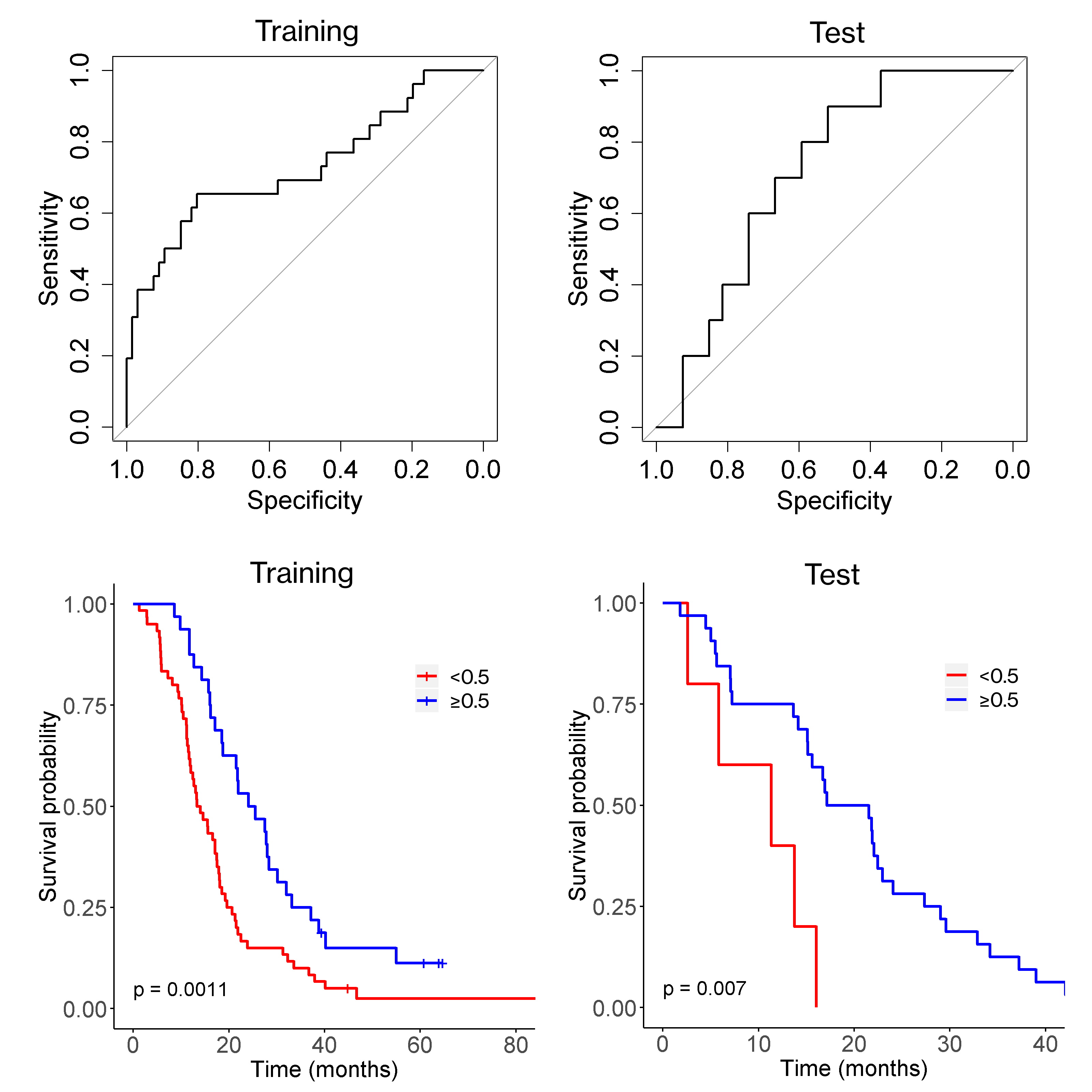


**Supplementary Figure S6. Filtering of dynamic susceptibility contrast (DSC) curves in the enhancement mask. The perfusion curves from the volume of interest are filtered with a low-pass filter at 10 Hz cut-off and normalized between [-1,0]. The curves with a correlation higher than 3 with an inverted Gaussian shape are selected as curves that reflect the bolus arrival. This was set experimentally, checking the selected and discarded curves for all cases. Once the curves were filtered, the original non-normalized curve values were the ones analyzed.**


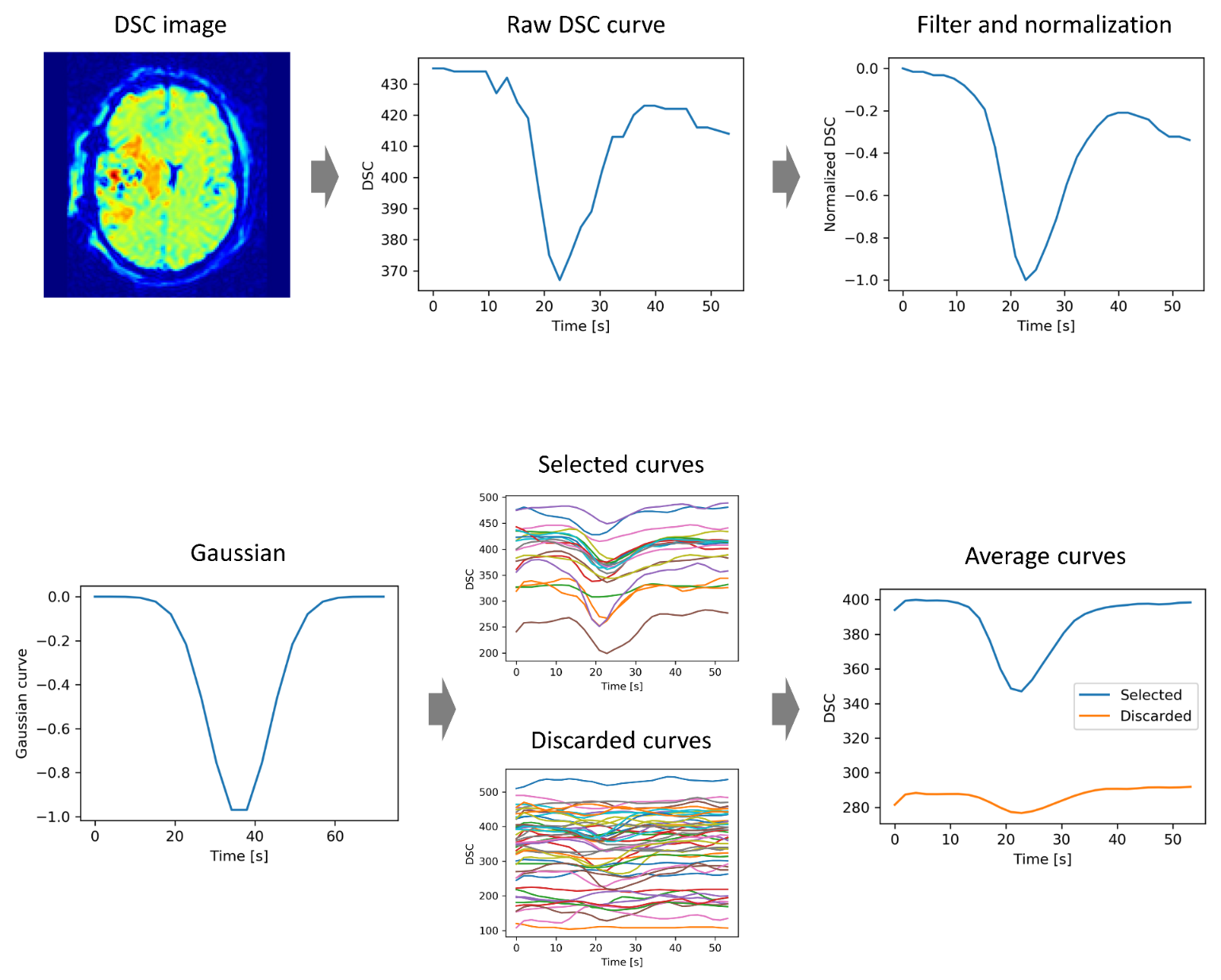


**Supplementary Table 1. Patient demographics and clinical data. Percentages are all referred to the indicated population. n = number of observations; SD = standard deviation; KPS = Karnofsky performance status; EPMR = early post-operative magnetic resonance.**

| **Category (unit)** | **Value (%)** |
| --- | --- |
| **Population (n)** |  |
| Primary GBM treated with Stupp | 144 |
|  |  |
| **IDH mutation** |  |
| No data | 89 (62) |
| IDH-wildtype | 53 (37) |
| IDH-mutant | 1 (1) |
|  |  |
| **Age (years)** |  |
| Median | 59 |
| Range | 20-77 |
|  |  |
| **Sex (n)** |  |
| Men | 92 (64) |
| Women | 52 (36) |
|  |  |
| **Survival (n)** |  |
| Non-censored | 129 (90) |
| Censored | 15 (10) |
|  |  |
| **Overall survival (months)** |  |
| Mean | 19.87 |
| SD | 13.77 |
| Median | 16.93 |
| Range | 1.27-84.57 |
| ≥2 years | 41 (28) |
| <2 years | 103 (72) |
|  |  |
| **Disease recurrence (n)** |  |
| Recurrence | 133 (92) |
| Progression free | 11 (8) |
|  |  |
| **Progression free survival (months)** |  |
| Mean | 11.60 |
| SD | 9.43 |
| Median | 9.07 |
| Range | 1.27-68.03 |
|  |  |
| **KPS** |  |
| Median | 80 |
| Range | 60-100 |
| KPS ≥ 90 (n) | 71 (49) |
| KPS < 90 (n) | 73 (51) |
|  |  |
| **Surgery to EPMR time (hours)** |  |
| Median | 48 |
| Mean | 62.67 |
|  |  |
| **Surgery to EPMR groups (n)** |  |
| 24 h | 26 (18) |
| 24-48 h | 51 (36) |
| 48-72 h | 42 (29) |
| 72-96 h | 12 (8) |
| 96-120 h | 6 (4) |
| 120-144 h | 5 (4) |
| 144-168 h | 2 (1) |

**Supplementary Table S2. Results of the Kaplan-Meier analyses for 99th percentile rCBV and maximum PSR in the entire population and in subgroups accounting for the time elapsed from surgery to the MRI scan. P-values were adjusted for multiple test comparison. n = number of observations; SD = standard deviation; rCBV = relative cerebral blood volume; PSR = percentage of signal recovery; h = hours.**

| **Variable** | **Group** | **Distribution** | | | **Kaplan-Meier survival curves** | |
| --- | --- | --- | --- | --- | --- | --- |
|  |  | **n** | **Mean** | **SD** | **Threshold** | **p-value** |
| **rCBV-99** | All | 113 | 8.02 | 4.44 | 8.26 | 0.050 |
|  | <24 h | 22 | 6.59 | 3.06 | 4.98 | 0.85 |
|  | 24-48 h | 37 | 7.63 | 4.06 | 7.93 | 0.004 |
|  | 48-72 h | 33 | 5.77 | 9.11 | 8.74 | 0.07 |
|  | ≥72 h | 21 | 8.47 | 3.61 | 9.96 | 0.011 |
| **Maximum PSR** | All | 113 | 111.0 | 8.0 | 112 | <0.001 |
|  | <24 h | 22 | 109.0 | 8.0 | 114 | 0.37 |
|  | 24-48 h | 37 | 110.0 | 8.0 | 111 | <0.001 |
|  | 48-72 h | 33 | 112.0 | 9.0 | 115 | <0.001 |
|  | ≥72 h | 21 | 111.0 | 8.0 | 103 | 0.10 |

**Supplementary Table S3. Patient demographics and clinical data in the training and test sets. Percentages are all referred to the indicated population. Differences in the distributions of the sets are statistically tested for continuous (Mann-Whitney) and categorical variables (Fisher’s exact test), checking data balance. n = number of observations; SD = standard deviation; KPS = Karnofsky performance status; EPMR = early post-operative magnetic resonance.**

| **Category (unit)** | **Value (%) training set** | **Value (%) test set** | **p-value** |
| --- | --- | --- | --- |
| **Population (n)** |  |  |  |
| Primary GBM treated with Stupp | 92 | 37 |  |
|  |  |  |  |
| **IDH mutation** |  |  | 0.53 |
| No data | 61 (66) | 27 (73) |  |
| IDH-wildtype | 31 (34) | 10 (27) |  |
|  |  |  |  |
| **Age (years)** |  |  | 0.27 |
| Median | 60 | 58 |  |
| Range | 26-77 | 37-73 |  |
|  |  |  |  |
| **Sex (n)** |  |  | 1.0 |
| Men | 60 (65) | 24 (65) |  |
| Women | 32 (35) | 13 (35) |  |
|  |  |  |  |
| **Survival (n)** |  |  | 0.32 |
| Exitus | 87 (95) | 37 (100) |  |
| Censored (survival >2 years) | 5 (5) | 0 (0) |  |
|  |  |  |  |
| **Overall survival (months)** |  |  | 0.67 |
| Mean | 21.06 | 18.61 |  |
| SD | 15.03 | 11.47 |  |
| Median | 17.08 | 16.73 |  |
| Range | 1.27-84.57 | 1.80-42.50 |  |
| **Dichotomous (n)** |  |  | 1.0 |
| ≥2 years | 26 (28) | 10 (27) |  |
| <2 years | 66 (72) | 27 (73) |  |
|  |  |  |  |
| **Disease recurrence (n)** |  |  |  |
| Recurrence | 88 (100) | 36 (100) |  |
| Progression free | 0 (0) | 0 (0) |  |
|  |  |  |  |
| **Progression free survival (months)** |  |  | 0.75 |
| Mean | 12.88 | 11.33 |  |
| SD | 13.55 | 9.14 |  |
| Median | 8.83 | 8.90 |  |
| Range | 1.27-74.20 | 1.67-40.10 |  |
|  |  |  |  |
| **KPS** |  |  | 1.0 |
| Median | 80 | 80 |  |
| Range | 60-100 | 60-100 |  |
| **Dichotomous (n)** |  |  | 0.70 |
| KPS ≥ 90 (n) | 45 (45) | 16 (47) |  |
| KPS < 90 (n) | 47 (55) | 21 (53) |  |
|  |  |  |  |
| **Surgery to EPMR time (hours)** |  |  | 0.14 |
| Mean | 61.04 | 70.70 |  |
|  |  |  |  |
| **Surgery to EPMR groups (n)** |  |  | 0.35 |
| 24 h | 18 (20) | 4 (11) |  |
| 24-48 h | 33 (36) | 13 (35) |  |
| 48-72 h | 27 (30) | 10 (26) |  |
| 72-96 h | 7 (7) | 4 (11) |  |
| 96-120 h | 2 (2) | 4 (11) |  |
| 120-144 h | 4 (4) | 1 (3) |  |
| 144-168 h | 1 (1) | 1 (3) |  |

**Supplementary Table S4. Selected radiomics variables by minimum-redundancy-maximum-relevance and stepwise regression in the training set, along with the fitted logistic regression coefficients. GLCM = grey-level co-occurrence matrix; GLDM = grey-level dependence matrix; GLRLM = grey level run length matrix; GLSZM = grey level size zone matrix; NGTDM = neighboring grey tone difference matrix.**

| **Variable** | **Coefficient** |
| --- | --- |
| Intercept | -1.1173 |
| First-order 10Percentile | -1.9545 |
| GLDM Gray Level Non Uniformity | -2.6181 |
| NGTDM Coarseness | -1.1213 |
| GLRLM Run Percentage | -2.1151 |
| GLCM Joint Average | -1.9391 |
| First-order Median | 1.8362 |
| GLCM Difference Average | -2.7634 |
| GLDM Dependence Entropy | -2.2417 |
| GLDM Dependence Non Uniformity | -5.052 |
| GLSZM Small Area High Gray Level Emphasis | 2.6875 |
| GLCM Id | -3.7622 |
| GLRLM Run Length Non Uniformity | 7.0092 |

**Supplementary Table S5. Multivariate Cox regression model for survival analysis combining all imaging features (mean enhancement thickness, DSC, radiomics) and clinical data (age, postoperative KPS). The age and mean thickness remain as independent prognostic factors. Further comments about the prognostic value of the variables are provided in the discussion.**

| **Variable** | **Cox regression** | | |
| --- | --- | --- | --- |
|  | **HR** | **95% CI** | **p-value** |
| Mean Thickness | 1.75 | 1.00-3.09 | 0.052 |
| Age | 1.04 | 1.02-1.06 | 0.0004 |
| KPS | 0.98 | 0.97-1.00 | 0.19 |
| rCBV-99 | 1.01 | 0.98-1.04 | 0.54 |
| Maximum PSR | 7.10 | 0.26-192 | 0.24 |
| First-order 10Percentile | 0.63 | 0.29-1.40 | 0.26 |
| GLDM Gray Level Non Uniformity | 2.02 | 0.86-4.72 | 0.10 |
| NGTDM Coarseness | 1.03 | 0.73-1.46 | 0.86 |
| GLRLM Run Percentage | 1.68 | 0.70-4.03 | 0.24 |
| GLCM Joint Average | 1.77 | 0.72-4.36 | 0.21 |
| First-order Median | 1.73 | 0.64-4.69 | 0.28 |
| GLCM Difference Average | 1.50 | 0.54-4.15 | 0.44 |
| GLDM Dependence Entropy | 0.85 | 0.45-1.59 | 0.60 |
| GLDM Dependence Non Uniformity | 0.64 | 0.12-3.48 | 0.60 |
| GLSZM Small Area High Gray Level Emphasis | 0.61 | 0.22-1.65 | 0.33 |
| GLCM Id | 2.06 | 0.54-7.83 | 0.29 |
| GLRLM Run Length Non Uniformity | 1.00 | 0.12-8.13 | 0.96 |

**Supplementary Table S6. Radiomics features extracted for each volume of interest (VOI) (94 features) and the associated coefficient of variation and its standard deviation when changing the extraction parameters. CV = coefficient of variation; SD = standard deviation; GLCM = grey-level co-occurrence matrix; GLDM = grey-level dependence matrix; GLRLM = grey level run length matrix; GLSZM = grey level size zone matrix; NGTDM = neighboring grey tone difference matrix.**

| **Variable** | **CV bin width** | **CV SD bin width** | **CV slice spacing** | **CV SD slice spacing** |
| --- | --- | --- | --- | --- |
| First-order 10Percentile | 0 | 0 | 7 | 5 |
| First-order 90Percentile | 0 | 0 | 4 | 3 |
| First-order Energy | 0 | 0 | 4 | 4 |
| First-order Entropy | 3 | 2 | 6 | 17 |
| First-order Interquartile Range | 0 | 0 | 9 | 16 |
| First-order Kurtosis | 0 | 0 | 13 | 11 |
| First-order Maximum | 0 | 0 | 8 | 6 |
| First-order Mean | 0 | 0 | 8 | 16 |
| First-order Mean Absolute Deviation | 0 | 0 | 7 | 16 |
| First-order Median | 0 | 0 | 12 | 11 |
| First-order Minimum | 0 | 0 | 30 | 45 |
| First-order Range | 0 | 0 | 10 | 16 |
| First-order Robust Mean Absolute Deviation | 0 | 0 | 9 | 16 |
| First-order Root Mean Squared | 0 | 0 | 6 | 8 |
| First-order Skewness | 0 | 0 | 14 | 6 |
| First-order Total Energy | 0 | 0 | 4 | 4 |
| First-order Uniformity | 12 | 3 | 10 | 7 |
| First-order Variance | 0 | 0 | 7 | 9 |
| GLCM Autocorrelation | 10 | 17 | 17 | 20 |
| GLCM Cluster Prominence | 3 | 2 | 29 | 13 |
| GLCM Cluster Shade | 2 | 1 | 10 | 11 |
| GLCM Cluster Tendency | 1 | 1 | 14 | 18 |
| GLCM Contrast | 2 | 9 | 42 | 26 |
| GLCM Correlation | 2 | 2 | 14 | 21 |
| GLCM Difference Average | 1 | 2 | 20 | 14 |
| GLCM Difference Entropy | 11 | 6 | 26 | 22 |
| GLCM Difference Variance | 3 | 16 | 54 | 23 |
| GLCM Id | 15 | 6 | 16 | 21 |
| GLCM Idm | 26 | 12 | 18 | 20 |
| GLCM Idmn | 6 | 8 | 14 | 19 |
| GLCM Idn | 5 | 7 | 18 | 19 |
| GLCM Imc1 | 21 | 23 | 17 | 21 |
| GLCM Imc2 | 23 | 21 | 32 | 26 |
| GLCM Inverse Variance | 41 | 19 | 20 | 19 |
| GLCM Joint Average | 9 | 14 | 13 | 18 |
| GLCM Joint Energy | 16 | 11 | 66 | 22 |
| GLCM Joint Entropy | 9 | 17 | 17 | 22 |
| GLCM MCC | 12 | 19 | 33 | 23 |
| GLCM Maximum Probability | 30 | 13 | 49 | 22 |
| GLCM Sum Average | 9 | 14 | 13 | 18 |
| GLCM Sum Entropy | 2 | 5 | 17 | 25 |
| GLCM Sum Squares | 1 | 1 | 17 | 18 |
| GLDM Dependence Entropy | 8 | 21 | 7 | 16 |
| GLDM Dependence Non Uniformity | 7 | 17 | 11 | 10 |
| GLDM Dependence Non Uniformity Normalized | 13 | 8 | 10 | 7 |
| GLDM Dependence Variance | 35 | 14 | 23 | 18 |
| GLDM Gray Level Non Uniformity | 1 | 2 | 8 | 9 |
| GLDM Gray Level Variance | 1 | 1 | 7 | 9 |
| GLDM High Gray Level Emphasis | 10 | 16 | 16 | 16 |
| GLDM Large Dependence Emphasis | 27 | 13 | 20 | 20 |
| GLDM Large Dependence High Gray Level Emphasis | 14 | 18 | 21 | 19 |
| GLDM Large Dependence Low Gray Level Emphasis | 67 | 17 | 73 | 30 |
| GLDM Low Gray Level Emphasis | 63 | 16 | 41 | 21 |
| GLDM Small Dependence Emphasis | 50 | 12 | 11 | 18 |
| GLDM Small Dependence High Gray Level Emphasis | 37 | 19 | 15 | 14 |
| GLDM Small Dependence Low Gray Level Emphasis | 19 | 10 | 27 | 16 |
| GLRLM Gray Level Non Uniformity | 7 | 4 | 8 | 9 |
| GLRLM Gray Level Non Uniformity Normalized | 11 | 3 | 8 | 7 |
| GLRLM Gray Level Variance | 3 | 3 | 7 | 9 |
| GLRLM High Gray Level Run Emphasis | 10 | 16 | 16 | 16 |
| GLRLM Long Run Emphasis | 7 | 7 | 16 | 20 |
| GLRLM Long Run High Gray Level Emphasis | 24 | 18 | 16 | 20 |
| GLRLM Long Run Low Gray Level Emphasis | 53 | 15 | 45 | 23 |
| GLRLM Low Gray Level Run Emphasis | 57 | 15 | 38 | 20 |
| GLRLM Run Entropy | 5 | 5 | 8 | 18 |
| GLRLM Run Length Non Uniformity | 13 | 7 | 7 | 9 |
| GLRLM Run Length Non Uniformity Normalized | 13 | 17 | 7 | 9 |
| GLRLM Run Percentage | 10 | 16 | 6 | 8 |
| GLRLM Run Variance | 9 | 9 | 20 | 22 |
| GLRLM Short Run Emphasis | 9 | 16 | 6 | 9 |
| GLRLM Short Run High Gray Level Emphasis | 12 | 17 | 16 | 15 |
| GLRLM Short Run Low Gray Level Emphasis | 57 | 15 | 37 | 20 |
| GLSZM Gray Level Non Uniformity | 24 | 24 | 10 | 8 |
| GLSZM Gray Level Non Uniformity Normalized | 16 | 20 | 11 | 8 |
| GLSZM Gray Level Variance | 11 | 8 | 8 | 9 |
| GLSZM High Gray Level Zone Emphasis | 15 | 17 | 18 | 15 |
| GLSZM Large Area Emphasis | 66 | 27 | 82 | 25 |
| GLSZM Large Area High Gray Level Emphasis | 112 | 36 | 58 | 28 |
| GLSZM Large Area Low Gray Level Emphasis | 83 | 37 | 102 | 31 |
| GLSZM Low Gray Level Zone Emphasis | 30 | 22 | 27 | 16 |
| GLSZM Size Zone Non Uniformity | 25 | 19 | 15 | 14 |
| GLSZM Size Zone Non Uniformity Normalized | 33 | 18 | 15 | 20 |
| GLSZM Small Area Emphasis | 25 | 17 | 14 | 20 |
| GLSZM Small Area High Gray Level Emphasis | 20 | 20 | 17 | 14 |
| GLSZM Small Area Low Gray Level Emphasis | 40 | 21 | 39 | 19 |
| GLSZM Zone Entropy | 11 | 7 | 7 | 16 |
| GLSZM Zone Percentage | 58 | 17 | 10 | 17 |
| GLSZM Zone Variance | 67 | 29 | 78 | 27 |
| NGTDM Busyness | 17 | 9 | 21 | 19 |
| NGTDM Coarseness | 10 | 3 | 12 | 8 |
| NGTDM Complexity | 11 | 16 | 22 | 11 |
| NGTDM Contrast | 15 | 22 | 20 | 19 |
| NGTDM Strength | 14 | 17 | 21 | 13 |

**Supplementary Methods. Radiomics variability according to different extraction parameters**

The 94 radiomics variables were extracted from the enhancing map segmentation in two ways: first, fixing the resampling to 1mm isotropic voxel size and varying the bin width (10, 25 and 50); second, fixing the bin width to 10 levels and varying the slice spacing (1, 2 and 5 mm). Values were not scaled. The variability of every feature was measured with the coefficient of variation (CV) between extractions and averaging over all the patients.

The resulting average CV and standard deviation (SD) for all radiomics variables is shown in Supplementary Table S6.

When varying the bin width: first-order radiomics variables show an average CV of 0.84% and all having a CV below 10% except uniformity (CV uniformity=12.37%). Features from the GLCM matrices have an average CV of 10.91%, GLDM an average of 25.07%, GLRLM shows 18.88% on average, GLSZM an average of 39.68% and NGTDM has 13.47% on average.

When varying the slice spacing: first-order radiomics variables show an average CV of 9.38%. Features from the GLCM matrices have an average CV of 24.33%, GLDM an average of 20.70%, GLRLM shows 16.38% on average, GLSZM an average of 31.98% and NGTDM has 18.99% on average.

The variability of features can be categorized as small (CV≤10%), intermediate (10%<CV≤20%) and large (CV>20%) [2]. A number of radiomics features showed small variability when changing the extraction parameters, and they seem more susceptible to the slice spacing than to the bin width. As expected, first-order features show smaller variability due to their global nature (they do not take into account the spatial distribution of voxels). Features derived from GLRLM and NGTDM matrices show intermediate variability, while GLCM, GLDM and GLSZM go from intermediate to high variability.

Both quantizing the intensity levels into bins and resampling the image spacing can affect the transitions of intensity from voxel to voxel and, therefore, the textural features. A sensible bin width and resampling with respect to the intensity levels and size of the areas of interest will reduce noise without removing relevant image contrast. In this regard, previous studies recommend a low bin width (from 1 to 20) and resampling to 1mm isotropic voxels [3,4].

References

1 Ellingson, B. M. *et al.* Validation of postoperative residual contrast-enhancing tumor volume as an independent prognostic factor for overall survival in newly diagnosed glioblastoma. *Neuro-Oncology* **20**, 1240-1250, doi:10.1093/neuonc/noy053 (2018).

2 Kim, H. *et al.* Impact of Reconstruction Algorithms on CT Radiomic Features of Pulmonary Tumors: Analysis of Intra- and Inter-Reader Variability and Inter-Reconstruction Algorithm Variability. *PLoS One* **11**, e0164924, doi:10.1371/journal.pone.0164924 (2016).

3 Duron, L. *et al.* Gray-level discretization impacts reproducible MRI radiomics texture features. *PLoS One* **14**, e0213459, doi:10.1371/journal.pone.0213459 (2019).

4 Molina, D. *et al.* Lack of robustness of textural measures obtained from 3D brain tumor MRIs impose a need for standardization. *PLoS One* **12**, e0178843, doi:10.1371/journal.pone.0178843 (2017).
